# Toxic Leadership in Emergency Nurses: Assessing Abusive Supervision and Its Team-Level Impacts on Conflict Management and Organizational Commitment

**DOI:** 10.1155/2024/4271602

**Published:** 2024-02-27

**Authors:** Nourah Alsadaan, Mohammed Alqahtani

**Affiliations:** ^1^College of Nursing, Jouf University, Sakaka 72388, Saudi Arabia; ^2^Department of Nursing, College of Applied Medical Sciences, King Faisal University, Alahsa, Saudi Arabia

## Abstract

**Background:**

Emergency departments suffer from authoritarian and manipulative leadership styles that affect team dynamics, emotional exhaustion, and quality patient care. However, little research specifically explores these toxic leadership effects on conflict management and nurses' organizational commitment.

**Objectives:**

This cross-sectional study aimed to assess the correlations between perceived toxic leadership, conflict resolution strategies, and commitment dimensions among emergency nurses while evaluating conflict tendencies as a mediating mechanism.

**Methods:**

A cross-sectional design that included multiple regression and mediation analyses was utilized. The sample consisted of 387 emergency nurses from five major Saudi hospitals surveyed using validated scales that measure perceived toxic leadership, conflict styles, and organizational commitment.

**Results:**

High prevalence rates for perceived authoritarian (77%), narcissistic (75%), and unpredictable (63%) leadership were reported. Increased toxicity was positively related to dominating and avoiding conflict styles but negatively related to integrating and compromising strategies. Toxic leadership is also associated with lower affective/normative commitment but higher continuance commitment. Conflict management partially mediated the leadership-commitment relationship, which explained 29% of the total effect. Finally, higher experience and education predicted greater perceived toxicity.

**Conclusions:**

The significant correlations between destructive leadership, adverse conflict, and reduced commitment in emergency nurses underscore the need for context-specific leadership training. Fostering supportive environments through multifaceted interventions can counteract toxicity impacts, impart constructive communication techniques, improve nurse well-being, and ensure high-quality patient care. As conflict tendencies and nurse characteristics influence susceptibility to detrimental leadership, tailored programs addressing experience levels are vital.

## 1. Introduction

Emergency departments (EDs) are critical high-stake environments within the healthcare system [1], where the pace is relentless and the margin for error is minimal [2, 3]. These units serve as the central hub for acute treatment, where decisions must be rapid and precise [4–6], with the potential to significantly alter patient outcomes [3]. The intensity and pressure inherent in emergency departments require a leadership style that not only facilitates rapid decision-making but also nurtures a supportive and cohesive team environment [7, 8]. However, the prevalence of authoritarian and manipulative leadership styles in such settings often undermines these objectives, introducing a toxic dynamic that can severely affect team functionality, staff well-being, and, ultimately, patient care quality [9–11].

Toxic leadership in emergency nursing is characterized by a spectrum of harmful behaviors [12, 13], including but not limited to abusive supervision, rigid top-down control [14], exploitation, self-serving actions, and emotional manipulation [15–17]. These behaviors collectively contribute to an environment where nursing staff can experience decreased well-being, reduced morale [18, 19], and a general sense of job dissatisfaction [20–23]. The extreme stress and urgency that define emergency care exacerbate the negative repercussions of such leadership, amplifying the challenges faced by nursing teams and compromising the quality of care provided to patients [24–26].

The literature extensively documents the adverse effects of toxic leadership in various organizational contexts, highlighting increased workplace stress, burnout, and emotional exhaustion among employees [27–29]. In the emergency nursing field, these effects are particularly pronounced due to the specialized nature of the work [30], which demands tight coordination among different specialists under intense pressure [31, 32]. Therefore, the vulnerability of emergency nursing teams to toxic leadership is markedly higher, given the critical reliance on effective communication, collaboration, and team cohesion to ensure optimal patient outcomes [33–35].

Despite the well-documented negative impacts of toxic leadership on organizational well-being and performance, there is still a significant gap in understanding how such leadership influences conflict confrontation strategies and organizational commitment among emergency nurses [36, 37]. This gap is particularly concerning given the critical importance of effective leadership in fostering team cohesion, maintaining high-performance standards [38–41], and ensuring the delivery of quality care in high-risk emergency settings [27–31]. Effective communication and the management of interpersonal tensions are crucial for maintaining constructive team dynamics [42, 43]. Yet, there is a dearth of research focusing on how emergency nurses, who are at the frontline of care delivery and coordination, navigate conflicts and maintain commitment in the face of toxic leadership [44–46].

Emergency nursing, with its unique challenges related to coordination, decision-making, and high-stakes outcomes, requires a leadership approach that supports rather than undermines team efforts [47, 48]. The distinct context of emergency care, where patient lives are frequently in the balance, underscores the nonnegotiable need for leadership that promotes rather than detracts from team cohesion, performance, and care standards [42, 49]. Therefore, there is a pressing need for research that specifically examines the repercussions of toxic leadership on emergency nurses [50, 51], particularly in terms of their strategies for confronting conflicts and their perceptions of organizational commitment [45, 52–56].

This study aims to address these critical gaps by providing an in-depth analysis of the impacts of toxic leadership on conflict resolution and organizational commitment among emergency nursing staff. By focusing on the unique challenges and dynamics of emergency nursing, the research seeks to uncover the specific ways in which toxic leadership behaviors manifest in this context and their implications for team dynamics, nurse well-being, and patient care. The ultimate goal is to inform targeted interventions and reforms that promote constructive communication and ethical leadership practices and support the well-being of nursing staff, thus improving the overall quality of emergency care delivery.

In doing so, this study not only aims to contribute to the existing body of knowledge on leadership in healthcare but also to provide practical insights that can guide the development of policies and practices to mitigate the negative effects of toxic leadership in emergency departments. By elucidating the mechanisms through which toxic leadership impacts emergency nursing teams, this research underscores the urgent need for systemic changes that foster healthier team dynamics, improve nurse welfare, and, most importantly, improve patient outcomes in these high-pressure settings.

## 2. Materials and Methods

### 2.1. Objective

1. Assess the prevalence of the perceived impact of toxic leadership behaviours experienced by emergency department nurses.
2. Investigate the relationship among the perceived impact of toxic leadership, organizational commitment, and conflict management strategies used by emergency department nurses.

### 2.2. Design

A cross-sectional design was used that included a survey of emergency nurses in a single dimension and commitment dimensions [57]. The ability to collect substantial descriptive numerical data from a relatively large sample to support quantitative statistical analysis was also an asset for investigating complex phenomena within emergency nursing environments [58]. Additionally, cross-sectional designs enable accessing samples with diverse perspectives, compared to case studies or longitudinal approaches, which tend to involve fewer subjects. Therefore, the methodology facilitated the evaluation of the impacts of perceived toxic leadership that can be generalizable in five major emergency departments [59]. Overall, the cross-sectional approach offered feasibility and generalizability advantages that aligned well with the study's objectives.

### 2.3. Settings

This study was completed in emergency care units of five large public hospitals in the northern province of Saudi Arabia. These hospitals provide emergency services to residents in major metropolitan areas, as well as smaller rural communities in the region. Emergency departments treat a high volume of patients annually and are staffed by nurses, physicians, specialists, and support personnel. The units operate 24/7 to provide critical and trauma care.

### 2.4. Sample

A systematic multistage sampling approach was adopted to select participants from large public emergency departments in a northern province. Initially, five hospitals were chosen according to predefined inclusion criteria. The target population was emergency department nurses who worked full-time and met inclusion criteria in five major public hospitals with emergency medicine departments in the Hail region of northern Saudi Arabia. These hospitals provide emergency and trauma services to urban and rural catchment areas. The parameters defining this population include their employment status (full-time registered nurses), primary workplace setting (emergency departments), and direct participation in patient care activities.

Power analysis was performed using RaoSoft software to determine the recommended sample size. The input included a 95% confidence level, a 5% error margin, an estimated population of nurses of 580 across sites, and a 50% response distribution. These parameters detected medium effect sizes between study variables with 80% power. The minimum adequate sample size calculated was 387 subjects. This aligned with the recommendations that nursing research should have sufficient power [60, 61].

The RaoSoft tool was chosen for its user-friendly interface and its ability to provide accurate sample size estimates based on essential criteria such as confidence level, margin of error, and expected distribution of responses [62]. Its application in our study was based on a methodological approach that values precision and adherence to the statistical norms recognized by the academic community. This approach ensures that our findings are robust and that the sample size adequately supports the study objectives and anticipated analyses.

A purposive, multistage sampling technique was utilized during recruitment at departmental meetings, through informational flyer distribution, and researcher visits. Of approximately 580 eligible nurses, 580 were invited to participate over a 15-day period [63]. Emphasizing voluntary participation and confidentiality during recruitment aimed to minimize sampling bias by encouraging representative enrollment across specialty experience levels.

Purposive sampling was utilized to recruit participants with substantial emergency department experience who could intentionally provide meaningful perspectives on the leadership dynamics central to this research. Although probability methods have advantages with respect to representativeness and generalizability, accessing informed participants was an efficient way to obtain rich insights into this complex phenomenon. During the multistage recruitment process, concerted efforts were made to emphasize voluntary participation and transparency around the purposes of the study to mitigate biases and approximate a representative enrollment of providers. However, the input to assess alternative probability sampling methods is valuable advice should subsequent follow-up studies be conducted to generalize findings further.

Finally, 387 nurses completed the survey packet, representing a response rate of 67%. This purposive approach supported accessing informed perspectives on a complex phenomenon from an adequately powered sample of experienced emergency department nurses. The sampled nurses reflected a diversity of ages, experience levels, genders, and education levels. Limitations include regional specificity since nurses were recruited from within a single Saudi Arabian province rather than nationally.

### 2.5. Eligibility Criteria

(i) Inclusion criteria:
  1. Registered nurses employed full-time in emergency departments.
  2. At least one year of experience in emergency care settings.
  3. Currently working in selected public hospitals in the northern province of Saudi Arabia.
  4. Provided informed consent to participate in the study.
(ii) Exclusion criteria:
  1. Nurses who took extended leave (e.g., maternity) in the last 6 months.

### 2.6. Data Collection Tools

Rigorously developed, psychometrically sound instruments were used to ensure a valid and reliable measurement of key variables, including perceived toxic behaviors, conflict tendencies, and organizational commitment. Extensive prior research provides confirmation of strong validity and reliability for the selected tools in various settings [64–68]. Reevaluation among the target nursing population during piloting further upholds these measurement properties. The following descriptions provide an overview of each established data collection tool, including interpretations.

1. Toxic Leadership Assessment [69]:
2. This 30-item scale developed by Schmidt (2008) measures perceived toxicity that spans five dimensions: abusive supervision, authoritarian leadership, narcissism, self-promotion, and unpredictability. Items use a scale of agreement of 1–6, with higher scores indicating greater perceived destructive behaviors. Previous studies in all fields produced Cronbach's alpha scores of 0.82–0.96 [65, 70], suggesting excellent internal reliability. The analysis of confirmation factors during the pilot test supported the multidimensional structure and validity of the construction among the target nurse population.
3. Rahim Organizational Conflict Inventory-II (ROCI-II) [67]:
4. Developed by Rahim in 1983, this validated inventory identifies the tendencies of conflict management style on five subscales: dominating, avoiding, obliging, compromising, and integrating. The 28 items reflect varying concerns for self-versus others when facing conflicts using a 5-point Likert scale. Extensive research reports that Cronbach's alpha reliability coefficients exceed 0.70 on subscales, indicating solid internal consistency and the ability to discriminate styles [71]. Pilot testing among nurses confirmed the reliability and stability of the factor structure.
5. Organizational commitment scale [72].
6. This instrument, conceptualized by Meyer and Allen (1991), assesses employee attachment across three commitment dimensions: affective, normative, and continuance. Using a 7-point Likert agreement format, higher scores reflect stronger feelings of emotional connection, perceived obligation, and necessity-driven commitment, respectively [72, 73]. Confirmatory research supports predictive validity regarding outcomes like turnover and performance [73]. Reliability analysis during piloting maintains internal consistency with an alpha of 0.88.

### 2.7. Ethical Approval

Ethical approval for this study was obtained from the Research Ethics Committee for Health Affairs in the Hail region, KSA (IRB: KACS, KSA: H-08-L-074). Permission to access the study sites and participants was granted by the nursing directors of the five participating emergency departments. Participants were provided with information sheets detailing the purpose of the study, voluntary participation, potential risks/benefits, and confidentiality measures. Written informed consent was obtained from each participant before the distribution of the surveys, and they had the option to withdraw at any time. The study followed ethical guidelines for nursing research according to the Declaration of Helsinki and the Code of Ethics of the International Council of Nurses, and the Ethics Committee reviewed all procedures and protocols to protect the rights and welfare of participants.

### 2.8. Procedure

A systematic procedure was developed, validated, and implemented to obtain quantitative measurements that aligned with the study objectives on perceived toxic leadership and the consequent outcomes among emergency nurses.

Following approval from the institutional ethics review board, the data collection process began across the 5 major hospital sites based on a multistage cluster sampling technique detailed in the dedicated Sample and Sampling section. The directors of the nursing department also provided site access approvals prior to recruitment.

The initial participation of the participants involved raising awareness through announcements at staff meetings and informational flyers on the importance of leadership dynamics research to evoke interest among emergency nurses. For nurses expressing interest and meeting the experience inclusion criteria, the principal investigator obtained written informed consent to participate after discussing:

- The purpose is to investigate perceived leadership behaviors and impacts.
- Survey response process and types of deidentified data collected
- Minimal risk and direct benefits associated with voluntary participation
- Right to withdraw participation anytime despite initial agreement
- Storage of completed surveys in an access-restricted locked cabinet

Consenting participants received numbered survey packets, including validated Likert-type scale instruments on perceived leadership toxicity (30 items), conflict confrontation tendencies (28 items), and organizational commitment (24 items), along with a basic demographic questionnaire. The packets had an estimated completion time of 15–20 minutes during work hours without disrupting the operation of the emergency department.

The sealed envelope collection boxes were placed in accessible common staff areas within each unit for returned packets over 2 consecutive weeks. Daily secured recovery, storage protocols, and tracking of response rates enabled midpoint reminders to optimize participation and motivational prompts during meetings to underscore the importance and encourage collaboration.

At the conclusion of the study, a participation rate of 67% was achieved, providing 387 fully completed nurse surveys for sufficient statistical power in the planned quantitative analyses. Encrypted data sets excluded any identifying details to uphold respondent rights and confidentiality standards governing ethical research. Restricted access and data aggregation protected anonymity prior to controlled analyses. This rigorous procedure allowed significant quantitative measurements aligned directly with the specific research questions on perceived toxic leadership outcomes.

### 2.9. Statistical Analysis

All data were analyzed using SPSS version 22.0 (IBM Corp, Armonk, NY). Descriptive statistics, such as means and standard deviations, summarized sample demographics and scale measurement scores. Pearson's correlation analysis quantified the bivariate relationships among perceived toxicity, conflict management styles, and organizational commitment types. Multiple linear regression examined predictors of affective commitment. The mediation analysis evaluated whether conflict management tendencies mediated the effect of destructive leadership on reduced commitment. For all tests, a *p* value <0.05 was considered statistically significant. Reliability analysis confirmed the internal consistency of measurement instruments. The assumptions of parametric testing were checked, including normality and homoscedasticity evaluation to meet the application criteria. The effect sizes were calculated to quantify the strengths of the observed relationship.

## 3. Results and Discussion

### 3.1. Results

The results of this cross-sectional study provide important insights into the complex relationships between perceived toxic leadership, conflict management approaches, and organizational commitment among emergency department nurses. Key findings demonstrate the high prevalence of destructive leadership behaviours reported by participants, with authoritarian, narcissistic, and unpredictable actions notably prevalent. Significant correlations emerged between perceived toxicity and conflict management styles, with passive and aggressive approaches positively associated but constructive strategies negatively related. Toxic leadership also related to lower affective/normative commitment but higher continuance commitment. Furthermore, conflict tendencies were found to partially mediate the link between destructive leadership and reduced commitment. Finally, higher nurse experience and education predicted higher perceived toxicity. The following sections delve deeper into these results, underscoring the need for supportive leadership and conflict training tailored to the intense emergency context. Elucidating these dynamics is the first step toward fostering healthy environments where nurses can thrive and provide optimal patient care even in high-stake situations.

Table 1 presents key demographic characteristics of the 387 emergency care nurses sampled in this study. The table shows a relatively young sample, with 83% under 40 years of age. Most were women (58%), typical for the nursing field. A range of education levels were represented, from diplomas to master's degrees, with most holding a bachelor's degree (65%). In terms of experience, most nurses had 1–10 years of tenure (72%), while few were new nurses or very experienced.

Table 2 effectively highlights the prevalence and characteristics of perceived toxic leadership behaviours among emergency nurse leaders. Inclusion of both the percentage of respondents reporting each behaviour and the mean score with standard deviations offers a nuanced understanding of the problem. The table shows that authoritarian leadership and narcissism are the most commonly perceived toxic behaviours, with 77% and 75% of nurses reporting these experiences, respectively, and correspondingly high mean scores (4.0 and 3.9). This suggests a significant impact of these behaviours on the workplace. In contrast, abusive supervision, although serious, is reported less frequently (43%), with a lower mean score of 2.3. The range of standard deviations (0.3 to 0.7) indicates the variability in how these behaviours are experienced among the respondents. The high prevalence rates, combined with notable mean scores, underscore the critical nature of addressing toxic leadership in emergency nursing settings to improve workplace dynamics and overall quality of care.

Table 3 of the study presents insightful findings on the relationship between various toxic leadership behaviours and organizational commitment, mediated by conflict management styles. The table reveals a negative correlation between all forms of toxic leadership and organizational commitment, indicating that higher levels of toxic behaviour's correspond to lower levels of commitment among emergency care nurses. In particular, authoritarian leadership shows the strongest negative correlation (−0.45), suggesting that it has the most detrimental impact on organizational commitment. The sizes of the mediation effect, ranging from 0.15 for self-promotion to 0.22 for authoritarian leadership, indicate that conflict management styles play a significant role in mediating these relationships. The statistical significance of these relationships is further underscored by the *p* values, with most falling below 0.001. These data imply that the way nurses manage conflict can significantly buffer or amplify the negative effects of toxic leadership on their commitment to the organization.

Table 4 presents the results of a mediation analysis that examined whether the conflict management style of nurses mediated the relationship between perceived toxic leadership and organizational commitment. The significant indirect effect (*B* = −0.33) suggests that conflict management partially mediates the association between perceived toxic leadership and commitment. This lends preliminary support to the hypothesis that leadership toxicity may influence nurses' conflict approaches, which in turn impacts their organizational commitment. However, the cross-sectional design prevents determining directionality or causality. The results should also be interpreted with caution given the reliance on subjective assessments of leadership toxicity. However, the table succinctly conveys that conflict management appears to play a mediating role in the link between perceived toxic leadership and organizational commitment among these emergency care nurses. The results provide initial evidence that the modification of conflict approaches could potentially counteract some negative impacts of toxic leadership on nurses' commitment.

Table 5 presents the results of a multiple linear regression model that predicts the affective organizational commitment of nurses to perceive toxic leadership, avoidant conflict style, integration of conflict style, age, and education level. The model was significant, which explained 28% of the variance in affective commitment. Higher perceived toxicity and avoidant conflict are independently related to lower affective commitment, while integrating style and age are associated with higher commitment. The large sample size lends confidence in the findings. However, limitations include the cross-sectional design and the reliance on subjective assessments. The variation of the common method can also inflate the relationships between the measured variables. However, the table succinctly summarizes key predictors of nurses' affective commitment, highlighting the negative impacts of destructive leadership and conflict avoidance, as well as the positive effects of collaborative conflict management.

Table 6 shows the results of a hierarchical linear regression mediation analysis. The significant indirect effect indicates that conflict management partially mediated the relationship between perceived toxic leadership and organizational commitment. Approximately 29% of the total effect of leadership on commitment operated indirectly through conflict management styles. This supports the hypothesis that toxic leadership may influence nurses' conflict approaches, which then affects their commitment. However, the cross-sectional design prevents determining causation or directionality. The subjective nature of leadership and conflict measures should also be considered. However, the table succinctly summarizes evidence that conflict management plays a mediating role in the association between perceived toxic leadership and commitment among these nurses. It points to the conflict style as a potential mechanism through which destructive leadership relates to reduced organizational commitment.

Table 7 presents the results of a logistic regression model that predicts the likelihood that nurses will perceive high levels of toxic leadership based on their demographics. Nurses with higher education levels and more years of experience showed significantly greater odds of reporting high toxicity. The model provides initial evidence that personal factors can affect perceptions and experiences of destructive leadership behaviours. However, the cross-sectional design prevents determining causality. Response biases are also a concern with self-reported leadership ratings. However, the table succinctly summarizes the exploratory findings suggesting that nurse education and tenure may predict perceived leadership toxicity, warranting further investigation. Although preliminary, the results point to potential vulnerabilities based on nursing background that could inform prevention efforts.

### 3.2. Discussion

This cross-sectional study aimed to address gaps regarding perceived toxic leadership impacts on conflict and commitment specifically in emergency nursing. The objectives were to assess the prevalence of the perceived impact of toxic leadership behaviors experienced by emergency department nurses and investigate the relationship between the perceived impact of toxic leadership, organizational commitment, and conflict management strategies used by emergency department nurses. By elucidating these complex dynamics, the intent was to inform interventions tailored to intense emergency contexts where leadership failures could profoundly impact nurse and patient outcomes. The following discussion interprets results regarding the stated objectives of delineating relationships among perceived toxic leadership, conflict management, and commitment in this understudied yet high-stake specialty setting.

Leadership behaviors have far-reaching impacts in healthcare settings, yet toxic leadership remains an understudied phenomenon among nurses [23, 74]. This concern is due to evidence linking detrimental leadership with poorer clinical outcomes, staff well-being, and quality care [75]. Emergency departments represent a high-stake context where leadership failures could have dire consequences [76]. However, little research has examined toxic leadership among emergency nurses specifically [23, 39, 77, 78]. This study helps address this gap by elucidating the impacts of perceived toxicity on the management of nurses' conflicts and organizational commitment. The findings promise to inform interventions to foster healthy leadership and optimal team functioning in this fast-paced environment where lives are on the line.

Our sample of 387 nurses from five emergency departments in Saudi Arabia reported high perceived toxicity, especially authoritarian, narcissistic, and unpredictable behaviours. Leadership toxicity was positively correlated with passive and aggressive conflict styles but negatively associated with constructive conflict approaches. Additionally, the increased perceived toxicity is related to a lower affective/normative commitment but a greater commitment to continued commitment. A salient finding was the mediating effect of conflict management, which explained nearly a third of the relationship between leadership and commitment. Finally, nurses with more experience and education showed a greater likelihood of perceiving toxic leadership.

#### 3.2.1. Prevalence of Toxic Leadership Behaviours

The high prevalence of perceived authoritarian (77%), narcissistic (75%), and unpredictable (63%) leadership behaviours confirm toxic leadership as an issue in emergency care settings. These rates exceed estimates from a recent meta-analysis that aggregates toxicity prevalence between industries (10–15%) [23]. The extreme stress and urgency of emergency departments can partially explain this elevated toxicity [7, 41]. However, the authors in [79] found lower perceived toxicity among Indian emergency nurses (14–23% for different behaviours). This discrepancy highlights the need for comparative data from multiple sites, as the organizational and cultural context can influence the prevalence. Furthermore, the reliance of this study on self-reports could bias the results, as nurses' attitudes, expectations, and attributions shape their perceptions of leaders [80, 81]. Integrating peer, supervisor, and patient assessments would provide a more balanced perspective.

#### 3.2.2. Conflict Management as a Mechanism

A significant finding was the mediating effect of conflict management, which explained almost a third of the total leadership-commitment relationship. This proposes conflict tendencies as a key mechanism that converts toxic leadership into attitudinal outcomes. Longitudinal and experimental studies could further validate this mediation model and directionality [82–84]. However, the results suggest that strengthening nurses' conflict management skills could potentially neutralize some detrimental impacts of poor leadership.

#### 3.2.3. Individual Susceptibility Factors

Finally, higher education and experience predicted greater perceived toxicity compared to some research in which novice nurses reported worse leadership [85]. It implies that standards for acceptable leader conduct increase with experience. Alternatively, toxic leaders can target underconfident junior nurses. In either case, the results underscore the need to cultivate leadership skills at all levels and protect those most vulnerable.

#### 3.2.4. Relationships among Toxic Leadership, Conflict Management, and Commitment

Significant positive correlations emerged between perceived toxic leadership and dominating/avoiding conflict styles, while negative associations with integrating and compromising approaches were observed. This is consistent with meta-analytic findings linking abusive supervision to more passive and aggressive conflict tendencies [86, 87]. However, contrasting reports show toxic leadership that specifically relates to avoiding rather than dominating conflict [23, 88]. This discrepancy could reflect situational factors, as the emergency context may require more dominant behaviors to match the urgency. Additionally, higher perceived leadership toxicity is related to lower affective/normative commitment but higher continuance commitment. These results support conclusions from a nursing review that identified leadership as an important determinant of organizational commitment [32, 89, 90]. However, the authors in [91] found no association between perceived toxicity and overall commitment among nurses. This discrepancy highlights potential cross-cultural variations in leadership-commitment dynamics.

#### 3.2.5. Mediating Role of Conflict Management

A significant finding was the mediating effect of conflict management on the toxic leadership-commitment relationship, which explained 29% of the total effect. This suggests that leadership behaviors can alter nurses' conflict approaches in a way that reduces commitment. Although scarce research has directly tested this mechanism, the authors in [92] similarly implicated conflict management as a mediator in the leadership-engagement relationship. However, the cross-sectional design prevents causal inferences. Longitudinal designs could help establish directionality.

#### 3.2.6. Practical Implications and Future Directions

The findings of this study underscore the critical need for the development of conflict management training programs specifically tailored for emergency department nursing staff. Such initiatives could potentially serve as a buffer against the detrimental effects of toxic leadership, potentially improving organizational commitment and workplace morale. Future research should extend these findings through longitudinal studies to determine the long-term impact of conflict management training, examine individual nurse characteristics that affect perceptions of leadership, and assess implications for patient care outcomes. Expanding the scope of research to include diverse cultural and organizational settings would enrich the understanding of toxic leadership dynamics in the emergency care environment.

## 4. Conclusions

This cross-sectional study provides valuable insights into the concerning prevalence of perceived toxic leadership behaviors among emergency department nurse leaders and the implications for nurses' conflict management approaches and organizational commitment. Key findings demonstrate high rates of authoritarian, narcissistic, and unpredictable leadership, which are positively associated with destructive conflict tendencies like domination and avoidance. In turn, these connect to lower affective/normative commitment. A salient finding is that conflict management explains almost a third of the total effect of toxic leadership on reduced commitment. The significant correlations found among toxic leader behaviours, adverse conflict strategies, and diminished commitment highlight the vital need to cultivate supportive leadership that fosters constructive communication and healthy team dynamics in intense, high-stake emergency departments. Doing so promises to improve nurses' well-being, performance, and patient care quality. The mediating effect of conflict management also suggests that this could be leveraged to mitigate detrimental leadership impacts.

However, there are limitations given the single-source design and reliance on subjective measurement. The cross-sectional methodology also prevents determining causality. Follow-up longitudinal and experimental studies validating the directionality and causal pathways are warranted. Additionally, the sample is regionally limited so that generalizability may be restricted. Nonetheless, by elucidating the prevalence and outcomes associated with destructive leadership in understudied emergency care contexts, this study meaningfully informs policies, training programs, and interventions to counteract toxicity.

## Figures and Tables

**Table 1:** Demographic characteristics of emergency care nurses (*n* = 387).

Variable	Category	*N*	%
*Age*			
	25–30 years	152	39
	31–40 years	170	44
	41–50 years	53	14
	>50 years	12	3

*Gender*			
	Male	162	42
	Female	225	58

*Marital status*			
	Single	127	33
	Married	152	39
	Divorced	75	19
	Widowed	33	9

*Education level*			
	Diploma	75	19.4
	Bachelor's degree	250	64.6
	Master's degree	62	16

*Years of experience*			
	1–5 years	180	47
	6–10 years	152	39
	11–15 years	37	10
	>15 years	18	4

**Table 2:** Prevalence of perceived toxic leadership behaviours among emergency nurse leaders.

Toxic leadership behaviour	*N*	%	Overall toxic leadership (M ± SD)
Self-promotion	212	55	2.8 ± 0.6
Abusive supervision	167	43	2.3 ± 0.7
Unpredictability	245	63	3.3 ± 0.5
Narcissism	289	75	3.9 ± 0.4
Authoritarian leadership	298	77	4.0 ± 0.3

**Table 3:** Relationship between toxic leadership and organizational commitment mediated by conflict management.

Toxic leadership behaviour	Correlation with organizational commitment	Mediation effect size	*p*
Self-promotion	−0.31	0.15	<0.01
Abusive supervision	−0.35	0.18	<0.01
Unpredictability	−0.40	0.20	<0.001
Narcissism	−0.42	0.21	<0.001
Authoritarian leadership	−0.45	0.22	<0.001

**Table 4:** Mediation analysis of the conflict management style on the relationship between toxic leadership and organizational commitment.

Path	*B*	SE	*p*
Toxic leadership in conflict management	0.57	0.08	<0.001
Toxic leadership to organizational commitment	−0.42	0.09	<0.001
Conflict management to organizational commitment	−0.33	0.07	<0.001

**Table 5:** Multiple linear regression analysis that predicts affective organizational commitment.

Variable	*B*	SE B	*β*	*p*
(Constant)	2.83	0.23		<0.001
Toxic leadership	−0.41	0.07	−0.32	<0.001
Avoidant conflict style	−0.38	0.09	−0.28	<0.001
Integrating conflict style	0.26	0.08	0.21	0.002
Age	0.02	0.01	0.14	0.012
Education level	−0.18	0.09	−0.11	0.048

**Table 6:** Mediation analysis using hierarchical linear regression.

Effect	*B*	SE B	*t*	*p*
Total effect				
Toxic leadership ⟶ commitment	−0.53	0.08	−6.98	<0.001
Direct effect				
Toxic leadership ⟶ commitment	−0.24	0.09	−2.73	0.007
Indirect effect				
Toxic leadership ⟶ conflict ⟶ commitment	−0.29	0.07	−4.25	<0.001

**Table 7:** Logistic regression predicts the likelihood of high toxic leadership based on nurse demographics.

Predictor	*B*	SE	Wald	*p*	Odds ratio
Age	0.02	0.04	0.21	0.646	1.02
Years of experience	0.18	0.09	3.92	0.048	1.20
Education level	0.67	0.28	5.73	0.017	1.95
Marital status	−0.34	0.20	2.88	0.090	0.71

## Data Availability

The data will be available on request.
